# Supplementary material for: 2023 Canadian Colposcopy Guideline: A Risk-Based Approach to Management and Surveillance of Cervical Dysplasia
Source: Curr Oncol. 2023 Jun 13;30(6):5738–68. doi: 10.3390/curroncol30060431 (PMC10297713; doi:10.3390/curroncol30060431)
Supplement: Supplementary file 1 [file curroncol-30-00431-s001.zip › curroncol-2420521-supplementary.pdf]

Supplemementary Table S1 . Search Strategy- Section 3.1 to 3.10

Conducted May 12, 2021

Ovid MEDLINE All

1	exp clinical pathway/
2	exp clinical protocol/
3	exp consensus/
4	exp consensus development conference/
5	exp consensus development conferences as topic/
6	critical pathways/
7	exp guideline/
8	guidelines as topic/
9	exp practice guideline/
10	practice guidelines as topic/
11	health planning guidelines/
12	(guideline or practice guideline or consensus development conference or consensus development conference, NIH).pt.
13	(position statement* or policy statement* or practice parameter* or best practice*).ti,ab,kf,kw.
14	(standards or guideline or guidelines).ti,kf,kw.
15	((practice or treatment* or clinical) adj guideline*).ab.
16	(CPG or CPGs).ti.
17	consensus*.ti,kf,kw.
18	consensus*.ab. /freq=2
19	((critical or clinical or practice) adj2 (path or paths or pathway or pathways or protocol*)).ti,ab,kf,kw.
20	recommendat*.ti,kf,kw.
21	(care adj2 (standard or path or paths or pathway or pathways or map or maps or plan or plans)).ti,ab,kf,kw.
22	(algorithm* adj2 (screening or examination or test or tested or testing or assessment* or diagnosis or diagnoses or diagnosed or diagnosing)).ti,ab,kf,kw.
23	(algorithm* adj2 (pharmacotherap* or chemotherap* or chemotreatment* or therap* or treatment* or intervention*)).ti,ab,kf,kw.
24	or/1-23 [CADTH CPG filter]
25	exp Uterine Cervical Dysplasia/
26	Cervical Intraepithelial Neoplasia/
27	Uterine Cervical Neoplasms/

28	((cervi* or vulva*) adj5 (dysplasia or neoplasia or neoplasm* or cancer* or carcinoma* or adenocarcinoma* or precancer* or pre cancer* or premalignan* or pre malignan* or preneoplas* or pre neoplas*)).ti,ab,kw,kf.
29	("cin 1" or cin1 or "cin 2" or cin2 or "cin 2+" or "cin2+" or "cin 3" or cin3).ti,ab,kw,kf.
30	(dyskaryosis or dyskariosis).ti,ab,kw,kf.
31	(((squamous adj5 (cell* or lesion*)) and (cervi* or vulva*)) or sil or lgsil or lsil or hgsil or hsil).ti,ab,kw,kf.
32	(ascus or "asc us" or "asc h").ti,ab,kw,kf.
33	(atypical glandular cell* or agc).ti,ab,kw,kf.
34	(abnormal* adj3 cervi*).ti,ab,kw,kf.
35	or/25-34
36	Early Detection of Cancer/
37	(screen* or detect* or prevent*).ti,ab,kw,kf.
38	or/36-37
39	35 and 38
40	((cervi* or vulva*) adj3 screen*).ti,ab,kw,kf.
41	39 or 40
42	Colposcopy/
43	colposcop*.ti,ab,kw,kf.
44	or/42-43
45	41 or 44
46	24 and 45

Supplementary Table S2. Search strategy- Section 3.12 (Equity in colposcopy)
Conducted Feb 7, 2022

Ovid MEDLINE(R) ALL <1946 to February 04, 2022>

#	**Query**	**Results**
1	exp Uterine Cervical Dysplasia/	4610
2	Cervical Intraepithelial Neoplasia/	10708
3	Uterine Cervical Neoplasms/	80383
4	((cervi* or vulva*) adj5 (dysplasia or neoplasia or neoplasm* or cancer* or carcinoma* or adenocarcinoma* or precancer* or pre cancer* or premalignan* or pre malignan* or preneoplas* or pre neoplas*)).ti,ab,kw,kf.	101067
5	("cin 1" or cin1 or "cin 2" or cin2 or "cin 2+" or "cin2+" or "cin 3" or cin3).ti,ab,kw,kf.	4786
6	(dyskaryosis or dyskariosis).ti,ab,kw,kf.	426

7	(((squamous adj5 (cell* or lesion*)) and (cervi* or vulva*)) or sil or lgsil or lsil or hgsil or hsil).ti,ab,kw,kf.	25981
8	(ascus or "asc us" or "asc h").ti,ab,kw,kf.	2521
9	(atypical glandular cell* or agc).ti,ab,kw,kf.	3360
10	(abnormal* adj3 cervi*).ti,ab,kw,kf.	3680
11	or/1-10	135244
12	Early Detection of Cancer/	32049
13	(screen* or detect* or prevent*).ti,ab,kw,kf.	4646898
14	or/12-13	4651939
15	11 and 14	45352
16	((cervi* or vulva*) adj3 screen*).ti,ab,kw,kf.	13546
17	15 or 16	46250
18	Colposcopy/	6692
19	colposcop*.ti,ab,kw,kf.	8968
20	or/18-19	10923
21	17 or 20	52487
22	exp Indigenous Canadians/	4098
23	(Indigenous or first nation* or metis or inuit or aboriginal or native).ti,ab,kw,kf.	280325
24	exp African Continental Ancestry Group/	93151
25	(Black or Blacks or African American* or African Canadian* or African Nova Scotian*).ti,ab,kw,kf.	210654
26	Rural Population/	65870
27	Rural Health/	23800
28	(rural or remote).ti,ab,kw,kf.	236520
29	exp Socioeconomic Factors/	484434
30	(low income or socioeconomic or socio-economic or poverty).ti,ab,kw,kf.	204373
31	exp "Emigrants and Immigrants"/	14439
32	Refugees/	11864
33	(immigrant* or newcomer* or refugee*).ti,ab,kw,kf.	40400
34	exp "Sexual and Gender Minorities"/	11526
35	bisexuality/ or exp homosexuality/ or transsexualism/	36552
36	(homosexual* or gay or gays or lesbian* or LGB* or bisexual* or queer or "sexual and gender minorit*" or sexual minorit* or men who have sex with men or women who have sex with women or two-spirit* or gender minorit* or genderqueer or intersex* or transgender* or trans female or trans male or transman or trans man or transmen or trans men or transpeople or trans people or transwoman or trans woman or transwomen or trans women or transsexual*).ti,ab,kw,kf.	54407
37	Language/	46643
38	language*.ti,ab,kw,kf.	185950
39	or/22-38	1543440
40	(barrier* or challenge* or delay* or timely or timeliness or non-attend*).ti,ab,kw,kf.	1666696

41	21 and 39 and 40	1228

Supplementary Table S3

Grade of Recommendations and Evaluation of Quality

**Table 1: Strength and Quality of Evidence Grades**	
Grade	Definition
Strength of Recommendation	
Strong	High level of confidence that the desirable effects outweigh the undesirable effects (strong recommendation for) or the undesirable effects outweigh the desirable effects (strong recommendation against)
Conditional/Weak	Desirable effects probably outweigh the undesirable effects (weak recommendation for) or the undesirable effects probably outweigh the desirable effects (weak recommendation against)
**Quality of evidence**	
High	We are very confident that the true effect lies close to that of the estimate of the effect.
Moderate	We are moderately confident in the effect estimate: The true effect is likely to be close to the estimate of the effect, but there is a possibility that it is substantially different.
Low	Our confidence in the effect estimate is limited: The true effect may be substantially different from the estimate of the effect.
Very Low	We have very little confidence in the effect estimate: The true effect is likely to be substantially different from the estimate of effect.

Adapted from GRADE handbook table 5.1

**Table 2: Implications of strong and weak recommendations for different users of guidelines**		
	Strong Recommendation	Weak Recommendation
**For patients**	Most individuals in this situation would want the recommended course of action and only a small proportion would not.	The majority of individuals in this situation would want the suggested course of action, but many would not.
**For clinicians**	Most individuals should receive the recommended course of action. Adherence to this recommendation according to the guideline could be used as a quality criterion or performance indicator. Formal decision aids are not likely to be needed to help individuals make decisions consistent with their values and preferences.	Recognize that different choices will be appropriate for different patients, and that you must help each patient arrive at a management decision consistent with her or his values and preferences. Decision aids may well be useful helping individuals making decisions consistent with their values and preferences. Clinicians should expect to spend more time with patients when working towards a decision.
**For policy makers**	The recommendation can be adapted as policy in most situations including for the use as performance indicators.	Policy making will require substantial debates and involvement of many stakeholders. Policies are also more likely to vary

		between regions. Performance indicators would have to focus on the fact that adequate deliberation about the management options has taken place.

Adapted from GRADE handbook table 6.1

Grade Handbook available at: https://gdt.gradepro.org/app/handbook/handbook.html
